# ABR, a novel inducer of transcription factor C/EBPα, contributes to myeloid differentiation and is a favorable prognostic factor in acute myeloid leukemia

**DOI:** 10.18632/oncotarget.22093

**Published:** 2017-10-26

**Authors:** Carolina Yaeko Namasu, Christiane Katzerke, Daniela Bräuer-Hartmann, Alexander Arthur Wurm, Dennis Gerloff, Jens-Uwe Hartmann, Sebastian Schwind, Carsten Müller-Tidow, Nadja Hilger, Stephan Fricke, Maximilian Christopeit, Dietger Niederwieser, Gerhard Behre

**Affiliations:** ^1^ Division of Hematology and Oncology, University Hospital Leipzig, Leipzig, Germany; ^2^ Division of Dermatology and Venereology, University Hospital Halle, Halle, Germany; ^3^ Division of Hematology and Oncology, University Hospital Heidelberg, Heidelberg, Germany; ^4^ Fraunhofer Institute for Cell Therapy and Immunology, Leipzig, Germany; ^5^ Department of Stem Cell Transplantation, University Medical Center Hamburg-Eppendorf, Hamburg, Germany

**Keywords:** acute myeloid leukemia, ABR, C/EBPα, myelopoiesis, prognostic

## Abstract

Active *BCR* related (*ABR*) gene deactivates ras-related C3 botulinum toxin substrate 1 (RAC1), which plays an essential role in regulating normal hematopoiesis and in leukemia. *BCR* gene, closely related to ABR, acts as a tumor suppressor in chronic myeloid leukemia and has overlapping functions with *ABR*. Evidence for a putative tumor suppressor role of *ABR* has been shown in several solid tumors, in which deletion of ABR is present. Our results show downregulation of *ABR* in AML. A block of ABR prevents myeloid differentiation and leads to repression of the myeloid transcription factor C/EBPα, a major regulator of myeloid differentiation and functionally impaired in leukemia. Conversely, stable overexpression of ABR enhances myeloid differentiation. Inactivation of the known ABR target RAC1 by treatment with the RAC1 inhibitor NSC23766 resulted in an increased expression of C/EBPα in primary AML samples and in AML cell lines U937 and MV4;11. Finally, AML patients with high *ABR* expression at diagnosis showed a significant longer overall survival and patients who respond to azacitidine therapy showed a significant higher ABR expression. This is the first report showing that *ABR* expression plays a critical role in both myelopoiesis and AML. Our data indicate the tumor suppressor potential of *ABR* and underline its potential role in leukemia therapeutic strategies.

## INTRODUCTION

Acute myeloid leukemia (AML) in adults is still associated with poor outcome [1]. AML involves activation of oncogenes or deactivation of tumor suppressor genes, and a block of differentiation caused by reduced function of transcription factors [2, 3]. Active BCR related (ABR) protein shares high homology with BCR (Breakpoint Cluster Region) [4], which acts as a tumor suppressor in chronic myeloid leukemia [5] and meningiomas [6]. Previous studies have reported a putative tumor suppressive role of *ABR* in several solid tumors, such as medulloblastoma [7], astrocytomas [8], and breast cancer [9] in which deletion of *ABR* has been found. Furthermore, ABR knockdown inhibited apoptosis and promoted colony formation from dissociated human embryonic stem cells [10]. *ABR* gene is ubiquitously expressed in mouse tissues, including bone marrow [11]. In mouse cells of the innate immune system, such as neutrophils and macrophages, the lack of a functional ABR protein results in abnormal reactivity of the innate immune system [11, 12]. ABR and the relative BCR specifically act as negative regulators of RAC1, which has been found to be active in myeloid-associated diseases [13–16] and plays crucial roles in many functions of the innate immune system [17]. Importantly, a previous study has found that RAC1 protein was overexpressed in AML patients [16]. Despite the known role of BCR in leukemia, there have been no reports showing any specific function of ABR in myeloid differentiation and AML.

The transcription factor C/EBPα is a major regulator of myeloid differentiation and functionally impaired in leukemia [18]. Furthermore, C/EBPα deficient hematopoietic cells do not effectively generate granulocyte-monocyte progenitors from the common myeloid progenitor [19, 20]. C/EBPα is also able to activate promoters of receptors for the granulocyte- (*G-CSF*) and monocyte-colony stimulating factor (*M-CSF*) [21, 22]. Interestingly, a previous study reported that M-CSF induced ABR activity in mouse bone marrow macrophages [23]. However, until now there is no evidence of a functional involvement of ABR in C/EBPα-dependent mechanisms in myelopoiesis. For this reason, we investigated the role of ABR in myelopoiesis and AML.

In this study, we examined the *ABR* expression pattern during normal and malignant myelopoiesis. A high *ABR* expression at diagnosis was associated with a significant longer overall survival of patients with *de novo* AML in complete remission which received hematopoietic stem cell transplantation. Furthermore, AML patients who responded to an azacitidine-based therapy showed a significant higher *ABR* expression than patients which did not respond. We also investigated the function of ABR in connection to C/EBPα. SiRNA-mediated block of ABR expression represses C/EBPα protein expression and prevents myeloid differentiation. In addition, we demonstrate that *ABR* induces the expression of C/EBPα as well as *M-CSFR*, *G-CSFR* and *miR-223*, which has been shown by our group and others as direct target of C/EBPα in granulopoiesis [21, 22, 24–26]. Finally, the inactivation of the known ABR target RAC1 by treatment with the RAC1 inhibitor NSC23766 resulted in an increased expression of C/EBPα. Altogether, our data present the first evidence that ABR acts as new enhancer of C/EBPα mRNA and protein levels and as favourable prognostic factor in AML.

## RESULTS

### ABR mRNA expression is repressed in acute myeloid leukemia (AML) and high ABR expression associates with improved outcome

**Figure 1:**
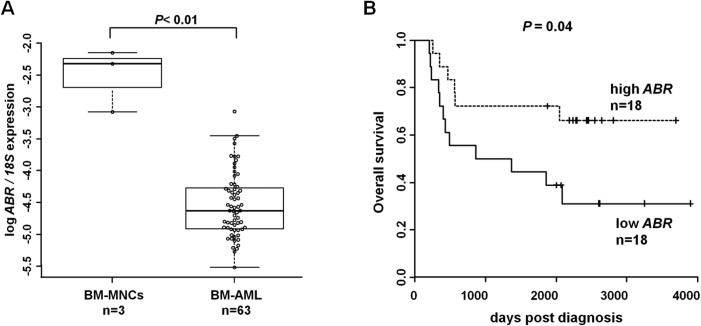
ABR expression is downregulated in acute myeloid leukemia (AML) and high ABR expression is associated with improved outcome (**A**) qPCR for ABR was carried out using bone marrow cells derived from AML patients at diagnosis (*n =* 63). Values were normalized to 18S and further to the expression level of bone marrow mononuclear cells (BM-MNCs) from healthy donors (*n =* 3). (**B**) Overall survival of the de novo AML patients in complete remission who received HSC transplantation (*n =* 36) is presented in Kaplan–Meier plots. ABR expression levels are cut at median. Log rank test was used for statistical evaluation.

A previous study showed that BCR acts as a tumor suppressor in chronic myeloid leukemia [5] and meningiomas [6]. Additionally, evidence for a putative anti-oncogenic role of ABR has been shown in several solid tumors [9–11] and in t(8;21) AML [31]. By qPCR we ascertained the *ABR* expression pattern in bone marrow samples of 63 primary AML patients at diagnosis and 3 healthy control bone marrow samples (Supplementary Table 4). Our data showed that *ABR* expression is strikingly repressed in bone marrow of AML patients independent of the subtype (Figure 1A). A recent treatment approach for AML is the allogeneic hematopoietic stem cell transplantation after non-myeloablative conditioning (NMA-HSCT). Which uses a low dose of total body irradiation to enable older patients to undergo HSCT [27]. By using the median cut algorithm in a subset of 36 de novo AML patients who received NMA-HSCT transplantation in complete remission, we observed that patients with high *ABR* expression showed a significant longer overall survival than patients with low *ABR* expression (Figure 1B). We observed that high *ABR* expression in AML correlates with favorable clinical and molecular patient characteristics in AML, such as a significant lower percentage of blasts in peripheral blood (*P =* .006) and high expression of miR-181a (*P <* .001) as well as tendencies for a lower number of white blood cells (WBC) (*P =* .06), and lower number of blasts in bone marrow (*P =* .06) (Table 1). We did not observe a significant difference in the mutational status of the prognostic relevant myeloid genes *CEBPA* and *FLT3* between high and low ABR expressers (Supplementary Table 3). However, a low ABR expression was associated with mutated *NPM1* (*P =* .04).

**Table 1:** Comparison of clinical and molecular characteristics of AML patients who received NMA-HSC transplantation with high versus low ABR expression

Characteristic	Low ABR (*n* = 31)	High ABR (*n* = 32)	*P* value
Median WBC count, 10^9^/L (range)	8.1 (1–385)	4.6 (0.7–140)	0.06
Median PB blasts, % (range)	50 (2–97)	21 (0–97)	0.006
Median BM blasts, % (range)	80 (27–95)	60 (10–94)	0.06
NPM1 mutation	11 (37%)	4 (13%)	0.04
High miR-181 a-1 expression, *n* (%)	9 (29%)	23 (71%)	< 0.001
High miR-181 a-2 expression, *n* (%)	8 (26%)	24 (75%)	< 0.001

Abbreviations: WBC, white blood count; PB, peripheral blood; BM, bone marrow. The median expression value was used as a cut point. It was calculated based on the expression levels assessed by qPCR. *P*-values compare patients who have low ABR expression versus high ABR expression by log rank test.

### ABR is increased during myelopoiesis

**Figure 2:**
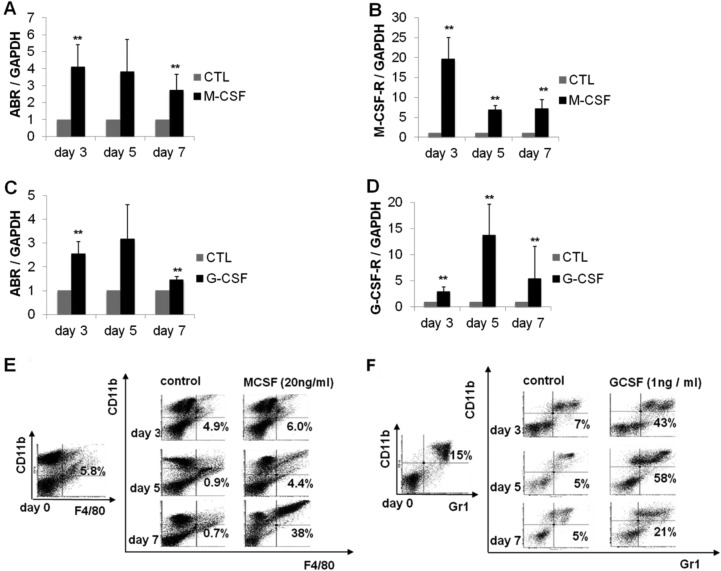
ABR expression is increased during myelopoiesis of mouse bone marrow cells Bone marrow cells derived from wild-type C57Bl/6 mice were treated with M-CSF or G-CSF for 7 days. (**A**–**D**) qPCR was carried out with oligonucleotides for ABR, M-CSF-R and G-CSF-R, respectively. Values were normalized to GAPDH and to the expression level of control treated cells (CTL). (**E**–**F**) FACS analysis was performed using CD11b and mouse specific macrophage (F4/80) and granulocyte (Gr1) antibodies. Total data are represented as mean ± SD from 3 representative experiments. ^**^P ≤ .01; ^*^P ≤ .05.

We could show that ABR expression is suppressed in AML in general. Furthermore, it is known that ABR protein shares M-CSF-induced activity with the related BCR protein in mouse bone marrow macrophages [23]. A previous study could show that C/EBPα has the capacity to direct monocytic development from myeloid progenitors [20]. In addition, C/EBPα is able to activate the M-CSF-receptor (M-CSF-R) promoter [21]. To access the role of ABR in myelopoiesis, we analysed ABR expression during myeloid differentiation of mouse bone marrow cells. The cells were cultured in the presence of M-CSF (20 ng/ml) or G-CSF (1 ng/ml) for seven days. Total RNA was isolated and quantified using qPCR. We could demonstrate that *ABR* expression was significantly increased upon M-CSF or G-CSF-stimulated differentiation of mouse bone marrow cells (Figure 2A–2B). The differentiation status of the cells was evaluated by qPCR based measurement of the monocytic marker M-CSFR and the granulocytic marker G-CSFR. The mouse bone marrow cells showed an induction of G-CSFR (up to 14 fold) and M-CSFR (up to 19 fold), respectively (Figure 2C–2D). Additionally, myeloid differentiation was assessed by flow cytometry using the myeloid surface marker CD11b and the mouse monocytic marker F4/80 and granulocytic marker Gr-1 (Figure 2E–2F).

### ABR contributes to myeloid differentiation

**Figure 3:**
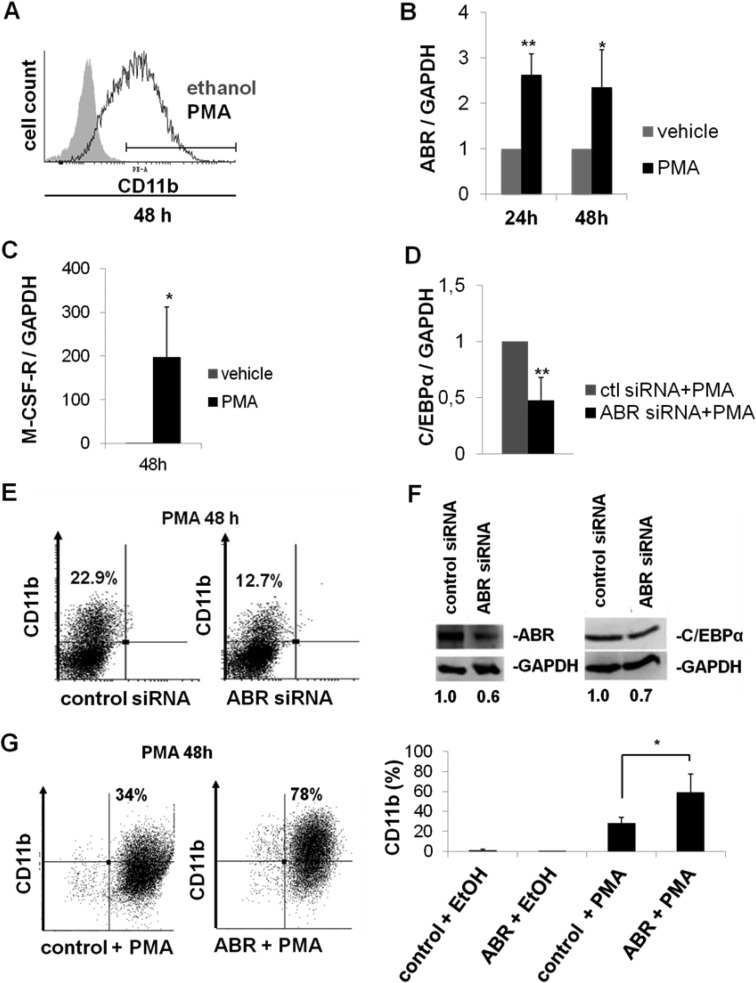
ABR promotes myeloid differentiation (**A**–**C**) U937 cells were treated with PMA (10nM) for the determined time points. (A) FACS analysis was performed using CD11b antibody. (B–C) Total RNA was analysed by qPCR with oligonucleotides for ABR (B) and M-CSF-R (C). Values were normalized to GAPDH and further to the expression level of control treated cells (vehicle). (**D**–**E**) U937 cells were transfected with ABR siRNA or control siRNA. Six hours after transfection, cells were stimulated with PMA (1nM) for 48 hours. (D) qPCR was carried out with oligonucleotides for C/EBPα. Values were normalized to GAPDH and to the expression level of control treated cells (ctl). (E) FACS analysis was performed using CD11b antibody. (**F**) U937 cells transfected with ABR siRNA or control siRNA. Total protein was analysed by western blot using ABR and C/EBPα antibodies. Values below the gel image indicate the protein levels normalized to GAPDH. (**G**) U937 cells were stably transfected with lentiviral vector EYFP-ABR in pCCL-cppt-MNDU3 and treated with PMA (10nM) for 48h. FACS analysis was performed using CD11b antibody. Total data are represented as mean ± SD from 3 independent experiments. ^**^P ≤ .01; ^*^P ≤ .05.

A previous study has shown that ABR is active upon MCSF stimulation in mouse bone marrow macrophages [23]. To further understand how the ABR activity is biologically significant in the context of myelopoiesis, we carried out differentiation experiments in U937 cells. U937 cells differentiate to macrophages during PMA (phorbol 12-myristate 13-acetate) induction [32]. The differentiation was confirmed by CD11b measurement (Figure 3A). By qPCR, we observed that ABR and M-CSF-R expression levels are increased significantly during differentiation (Figure 3B–3C). To further analyze the role of ABR in differentiation, we transfected U937 cells with ABR siRNA or control siRNA. Six hours after transfection, the cells were stimulated with 1nM PMA. We found that a block of ABR expression resulted in a 53% diminution of *CEBPA* expression levels after PMA treatment (Figure 3D). The flow cytometry analysis revealed that ABR knockdown in leukemic U937 cells leads to a significant reduction of the surface marker CD11b in comparison to the control siRNA transfected cells after PMA treatment (Figure 3E). ABR knockdown was verified by western blot and showed a 40% reduction of the ABR protein 24 hours after siRNA treatment in comparison to the control. Analysis of CEBPα protein revealed a 30% reduction of C/EBPα protein levels 24 hours after siRNA mediated knock down of ABR (Figure 3F). Conversely, lentiviral overexpression of ABR in U937 cells followed by treatment with PMA for 48 h showed a 2.3-fold increase in the CD11b positive cell population (Figure 3G). These findings illustrate a specific role of ABR in PMA induced monocytic differentiation.

### ABR induces C/EBPα expression and thereby increases the expression of M-CSF-R, G-CSF-R and miR-223

**Figure 4:**
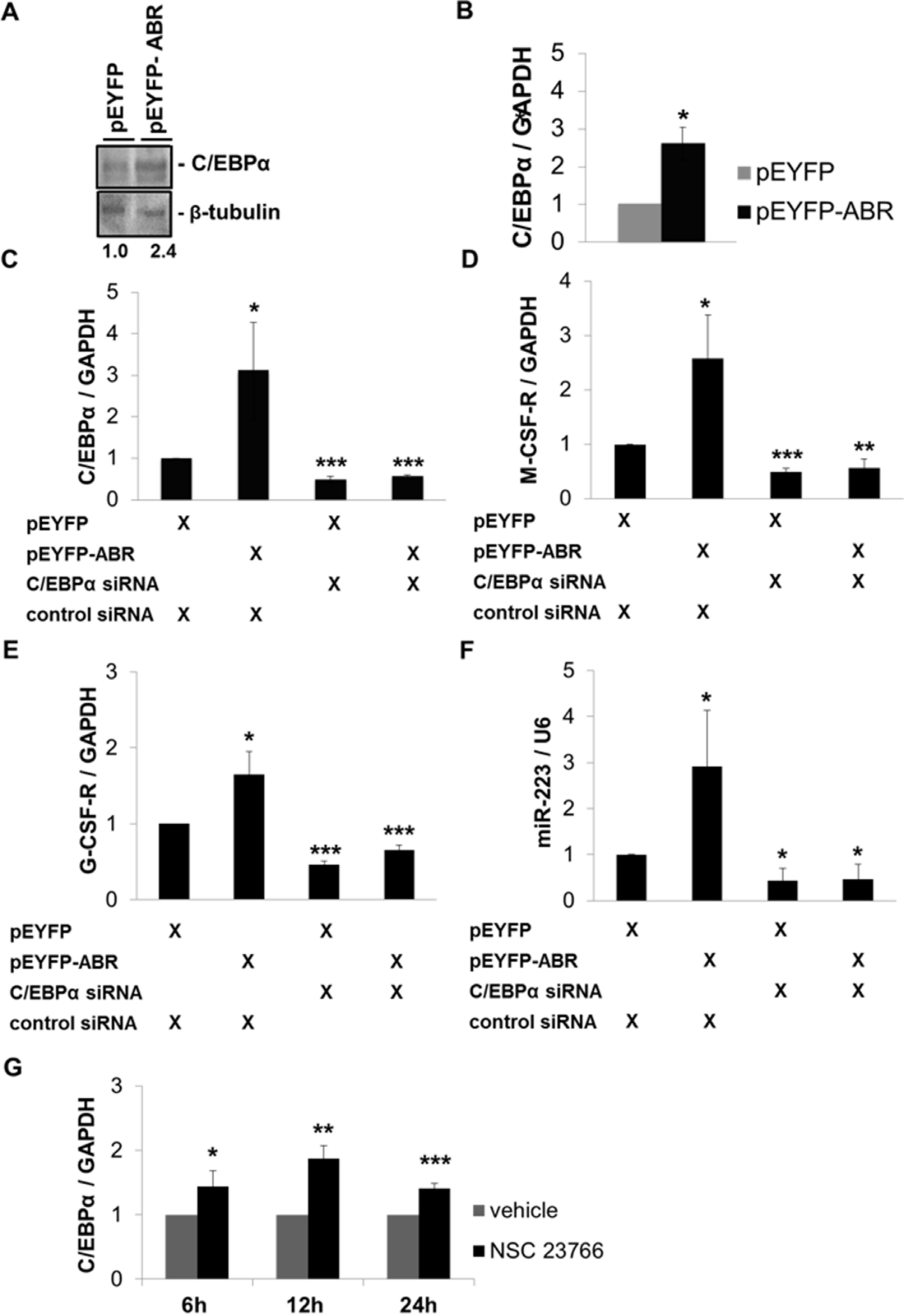
ABR induces C/EBPα expression and thereby increases the expression of M-CSF-R, G-CSF-R and miR-223 (**A**–**B**) U937 cells were transfected with ABR or empty vector for 24 hours. (A) Total protein was analysed by western blot with C/EBPα antibody. Values below the gel image indicate C/EBPα protein levels normalized to β-tubulin. (B) Total RNA was analysed by qPCR with oligonucleotides for C/EBPα. Values were normalized to GAPDH. (**C**–**F**) U937 cells were co-transfected with ABR or empty vector along with C/EBPα siRNA or control siRNA for 24 hours. Total RNA was analysed by qPCR with oligonucleotides for C/EBPα, M-CSF-R, G-CSF-R and miR-223. Values were normalized to GAPDH and to the expression level of control transfected cells. (**G**) U937 cell were treated with 100 μM Rac1 inhibitor NSC23766 for the indicated time points. Total RNA was analysed by qPCR with oligonucleotides for C/EBPα. Values were normalized to GAPDH and to the expression level of control treated cells (H_2_O). Total data are represented as mean ± SD from 3 independent experiments. ^***^P ≤ .001; ^**^P ≤ .01; ^*^P ≤ .05.

Because ABR knockdown reduced C/EBPα on mRNA and protein level, we next examined the ability of ABR to regulate the expression of C/EBPα. Thus, we performed transient overexpression of ABR and measured resulting C/EBPα protein levels. Here, we could demonstrate that enforced ABR expression for 24 hours leads to a 2.6-fold increase in C/EBPα mRNA and a 2.4-fold enhancement of C/EBPα protein in U937 cells (Figure 4A–4B). Conversely, the co-transfection of C/EBPα siRNA and ABR for 24 hours blocked ABR-mediated induction of *CEBPA* and its targets *G-CSF-R*, *M-CSF-R* and miR-223 as well (Figure 4C–4F). Interestingly, the inactivation of the known ABR target RAC1 by treatment with the RAC1 inhibitor NSC23766 resulted in a significant increase of *CEBPA* mRNA to 87% (Figure 4G). These data indicate a role of ABR in the C/EBPα regulation.

### Rac1 inhibitor induced C/EBPα expression in primary AML cells

**Figure 5:**
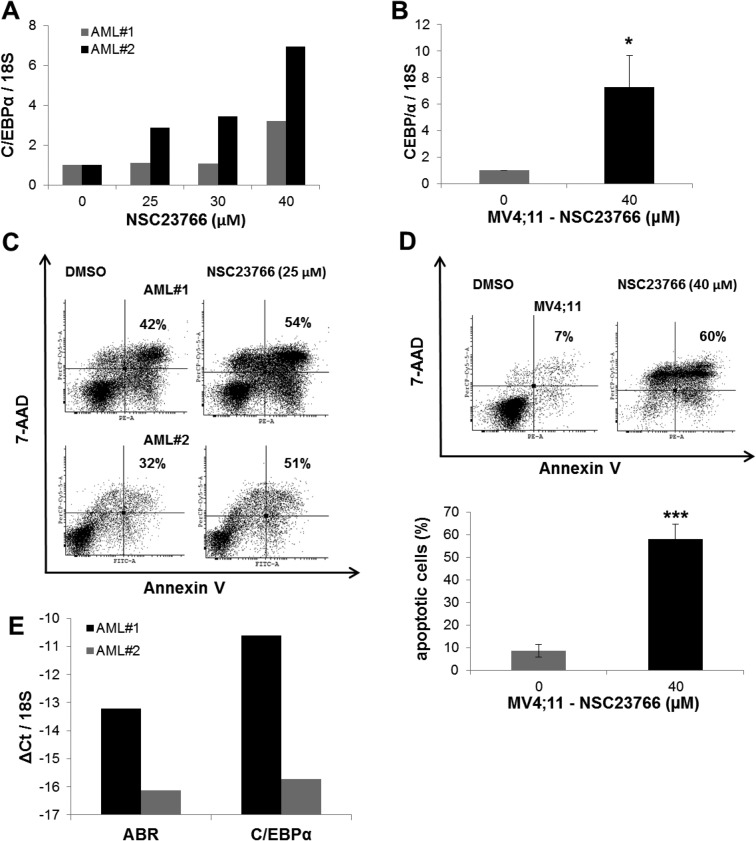
Rac1 inhibitor induced C/EBPα expression in AML primary cells (**A**–**B**) AML primary cells (A) or AML MV4;11 cells (B) were treated with Rac1 inhibitor NSC23766 using the indicated concentrations for 72 h (A) or 48h (B). (A–B; E) Total RNA was analysed by qPCR with oligonucleotides for C/EBPα. Values were normalized to 18S and to the expression level of control treated cells (DMSO). (**C**–**D**) Flow cytometry analysis for Annexin V and 7-AAD staining in AML primary cells (C) or MV4;11 cells (D) treated with NSC23766. (**E**) Total RNA was analysed by qPCR with oligonucleotides for ABR and C/EBPα. Values were normalized to 18S. Total data (B, D–E) are represented as mean ± SD from 3 independent experiments. ^***^P ≤ .001; ^**^P ≤ .01; ^*^P ≤ .05.

Because Rac1 inhibition increased *C/EBP*α expression, we investigated the effect of Rac inactivation in primary AML cells. To address this issue, we treated primary AML samples with the Rac1 inhibitor NSC23766. We could show that Rac1 inhibitor treatment induced the expression of C/EBPα up to 7 fold (Figure 5A) and enhanced the percentage of apoptotic cells up to 60% (Figure 5C). We also observed that leukemic MV4;11 cells exhibited a 7-fold increase in the expression of C/EBPα (Figure 5B). Furthermore, in MV4;11 cells NSC23766-treatment caused a 6-fold increase in apoptosis (Figure 5D). In addition, we analysed ABR expression in the primary AML samples. We found that AML sample #2 had a reduced ABR expression in comparison to AML sample #1. Interestingly, we observed that the effects of Rac inhibition with NSC23766 are enhanced in AML sample #2, which showed a 60% increase in the apoptotic cell number in comparison to AML sample #1, which had only 30% increase (Figure 5E).

### High ABR expression associates with response to azacitidine treatment in AML patients

**Figure 6:**
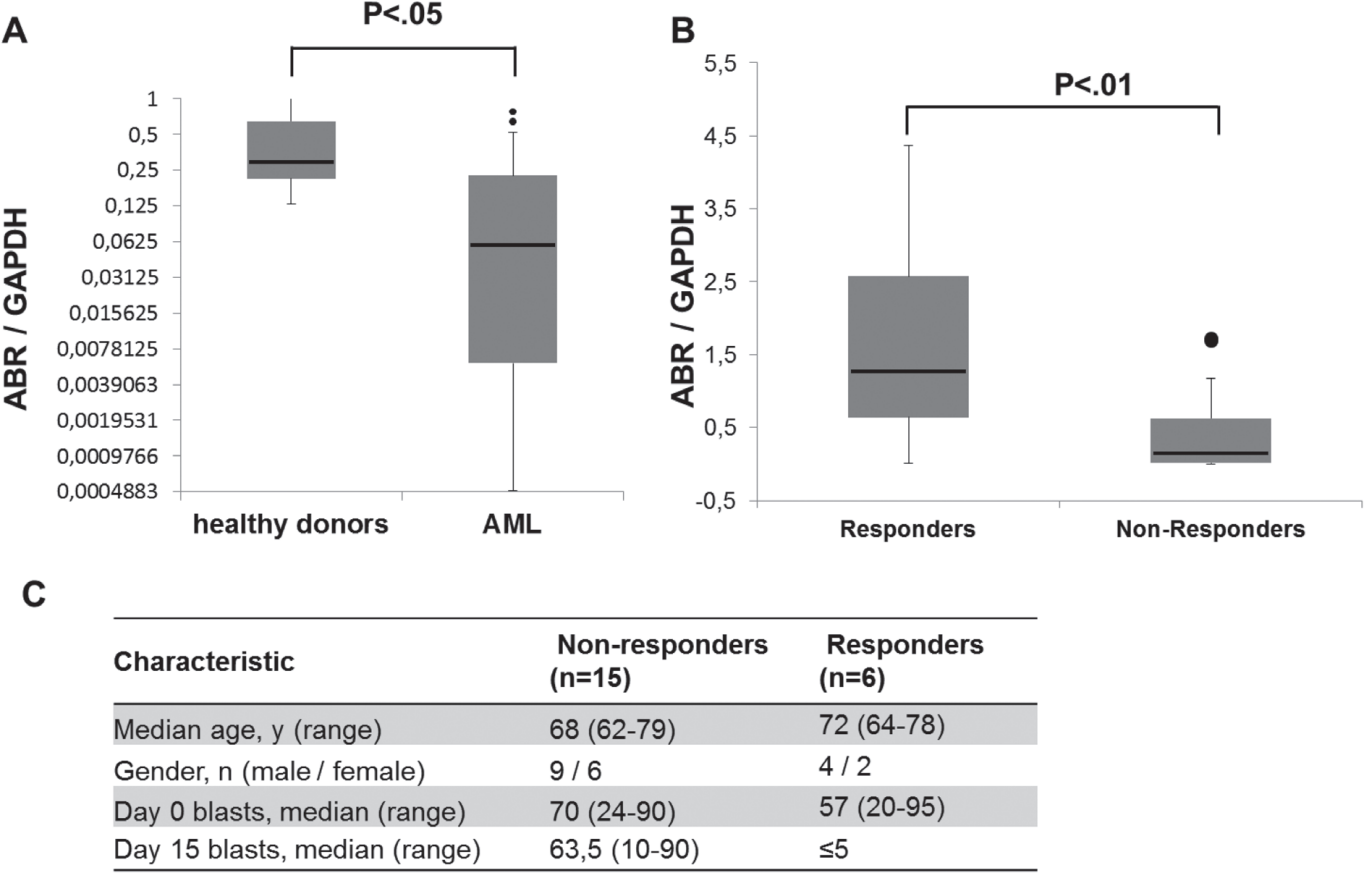
Response to azacitidine treatment in AML patients associates with high ABR expression (**A**–**B**) qPCR for *ABR* was carried out using bone marrow cells derived from AML patients at diagnosis (*n =* 21). Values were normalized to GAPDH and to the expression level of bone marrow mononuclear cells (BM-MNCs) from healthy donors (*n =* 3). Kruskal test was used for statistical evaluation. (**C**) Characteristics of azacitidine-treated AML patient samples used for *ABR* expression analysis.

Azacitidine is an inhibitor of DNA methylation that induces tumor suppressor gene expression [33]. As we hypothesize that *ABR* could act as a tumor suppressor gene in AML, we examined the *ABR* expression in azacitidine-treated AML patients (Supplementary Table 4). To this end, bone marrow samples from 21 AML patients (> 60 years) were collected at diagnosis and *ABR* expression was quantified by qPCR method (Figure 6A). Our data showed that *ABR* expression is repressed in bone marrow of AML patients at diagnosis in comparison to healthy control samples (Figure 6A). After azacitidine therapy, patients were divided into two groups (responders and non-responders). Response was defined as a blast clearance (≤5%) in the bone marrow on day 15 after start of the first azacitidine treatment cycle [28] which could be achieved in 11 of 21 patients (42%) (Figure 6C). We found that a high *ABR* expression significantly correlated with a better response after azacitidine treatment (Figure 6B). These observations suggest that *ABR* as a relevant target for the treatment of leukemia.

### ABR increases azacitidine-induced apoptosis

**Figure 7:**
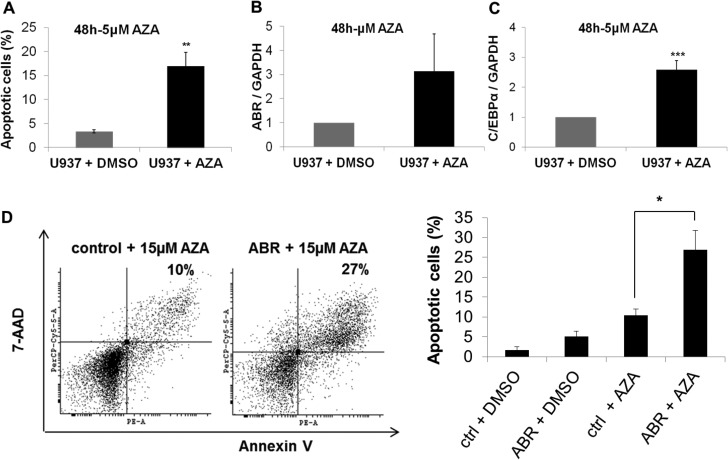
ABR overexpression enhances azacitidine-induced apoptosis (**A**–**B**) Flow cytometry analysis for Annexin V and 7-AAD staining in non-transduced U937 cells (A–C) or in U937 cells stably expressing ABR or control sequence (D) or in to analyse apoptosis. Cells were treated with 5 μM (A–C) or 15 μM (D) azacitidine (AZA) or DMSO for 48 hours. (**C**–**D**) qPCR analysis of *ABR* (B) and *CEBPA* (C) expression in non-transduced U937 cells after AZA treatment (5 µM). Total data are represented as mean ± SD from 3 representative experiments. ^**^P ≤ .01; ^*^P ≤ .05.

Because our data show that ABR induces the transcription factor C/EBPα and is a favorable prognostic factor in AML, we were interested in the impact of ABR on AML treatment. Azacitidine is one of the most effective DNA demethylating agents used in cancer treatment and widely used for the treatment of acute myeloid leukemia [34]. We could show that azacitidine is able to induce apoptosis in U937 cells (Figure 7A) and azacitidine alone leads to induction of the mRNA expression levels of *ABR* and *CEBPA* in U937 cells (Figure 7B–7C). To investigate the effect of ectopic ABR expression in azacitidine therapy, we stably overexpressed *ABR* in U937 cells and treated the cells with 15 µM azacitidine. ABR significantly enhanced the proapoptotic effect of azacitidine (2.7 fold with 15 µM azacitidine for 48 hours) in comparison to the control (Figure 7A). These results suggest that a putative epigenetic modification may be responsible for a reduced expression of *ABR* in AML.

## DISCUSSION

ABR deactivates the small GTPase Rac1, a master molecular switch that regulates several cellular processes, including maintenance and expansion of leukemic cells [16] and regulation of immune cell functions [17]. Previous studies of ABR have mainly focused on ABR involvement in immune regulation. Mice lacking ABR exhibit an increased susceptibility to several inflammatory diseases, demonstrating that ABR activity is required for appropriate control of innate immune response *in vivo* [12, 35, 36]. Furthermore, a connection between ABR deletion and several non-hematopoietic tumors has been reported [7–9]. These observations suggest that ABR is a relevant target for the treatment of leukemia and possibly of several other types of cancer. An important recent finding is that active RAC1 promotes expansion of leukemic stem cells by mediating their interaction with stromal cells in mouse AML-ETO-9a model [16, 37]. This finding is supported by other studies, which reported that RAC1 is required for the engraftment phase of hematopoietic reconstitution in mice [38, 39]. Similarly, MLL-AF9–transduced murine leukemic stem cells showed enhanced levels of active RAC [40]. Although the relative BCR is demonstrated to act as a tumor suppressor in leukemia [5], a specific function of ABR in myelopoiesis and leukemia has not been addressed. Therefore, in the present study, we utilize mouse bone marrow cells, leukemic cells and AML patient samples as models to investigate the role of ABR in myelopoiesis and leukemia. A previous report described a reduction of ABR expression in t(8;21) AML [31]. Indeed, our analyses show that *ABR* expression is strikingly reduced in AML among different subtypes (Figures 1A and 6A). Consistently, elevated levels of active RAC1, which is downregulated by ABR, have been previously described in primary human CD34 positive hematopoietic stem cells isolated from patients with AML [16]. Our study identifies a novel function of ABR, showing that ABR contributes to myeloid differentiation (Figure 3). By analyzing a subset from de novo AML patients, in complete remission which received HSC transplantation, we clearly see a prognostic correlation between a high *ABR* expression and a longer survival of the patients (Figure 1B). In line with this, we could demonstrate that AML patients with higher *ABR* expression levels respond better to clinical azacitidine-based therapy (Figure 6B). In accordance with this, we observed that high *ABR* expression at diagnosis is associated with favorable clinical and molecular patient characteristics, (Table 1), which have been shown to be correlated with favorable outcome in AML patients [41, 42]. A low *ABR* expression was associated with mutated *NPM1*. *NPM1* mutation in the absence of fms-like tyrosine kinase 3 internal tandem duplications (*FLT3-ITD*) is associated with a relatively favorable prognosis. However, *NPM1* is a late driver mutation [43], identified as being frequently overlapped with *FLT3-ITD*, which is associated with an unfavorable prognosis [44]. Moreover, the persistence of *NPM1* mutation was associated with a significantly higher risk of relapse [45].Those data suggest that *ABR* is a key mediator of tumor suppressor function in AML. Conversely, a reduced ABR expression correlated with a disturbance of myeloid differentiation (Figure 3D) and worse outcome in AML (Figures 1B and 6B). This study provides the first evidence that repression of *ABR* is a common phenomenon in AML with different subtypes (Figures 1A and 6A). Because RAC1 is downregulated by ABR [23], a reduction of *ABR* expression might contribute to the accumulation of RAC1 in AML. In fact, Rac1 is active, overexpressed in chronic and acute myeloid leukemia patients [14–16] and promotes leukemia development through enhancing leukemia cells’ homing and retention in niche [37]. Interestingly, we found that Rac1 inhibition with NSC23766 induced C/EBPα expression in primary AML cells and in AML cell lines U937 and MV4;11 (Figures 4G and 5A–5B). Rac1 is involved in regulating the phosphorylation of the signal transducer and activator of transcription 3 (STAT3) [46], a transcription factor activated by tyrosine kinases [47]. In acute myeloid leukemia, constitutive activation of STAT pathway has been reported in various studies [46, 48, 49]. In addition, phosphorylation of STAT3 results in the induction of c-myc proto-oncogene product (Myc) [46]. Several studies have shown that transcription factor Myc is able to negatively regulate C/EBPα expression and block C/EBPα transactivation function [50–52]. Consistently with those findings, Rac1 inhibition with siRNA or Rac1 inhibitor NSC23766 has been shown to repress Myc transcription [53]. Therefore it is tempting to speculate that ABR-mediated Rac inhibition might induce CEBPA gene transcription via Myc downregulation.

Azacitidine acts as a hypomethylating agent in myeloid malignancies [54] and is licensed for AML therapy [55]. We found that response to azacitidine treatment is associated with high *ABR* expression in elderly patients with AML (Figure 6). We could show that ABR enhances azacitidine-induced apoptosis (Figure 7A). These data highlight the role of ABR in therapy response. Additionally, azacitidine treatment increased the expression levels of *ABR* and CEBPα (Figure 7C–7D), suggesting that a potential epigenetic modification may be responsible for downregulation of ABR in AML. Further, it is shown that the inactivation of the known ABR target Rac1 in leukemia increases chemotherapy-induced apoptosis [37, 56], supporting a possible mechanism of ABR action in AML therapy via inhibition of Rac1. In line with this, the treatment of leukemic cells with the Rac1 inhibitor NSC23766 resulted in an increased expression of *CEBPA* (Figures 4G, 5A, 5B), suggesting that ABR might induce *CEBPA* expression via Rac1 repression.

C/EBPα functions as a key mediator of myelopoiesis, which is a key step disrupted in distinct subtypes of AML [57]. The receptors for G-CSF (G-CSF-R) and M-CSF (M-CSF-R) as well the miR-223 are direct targets of C/EBPα [21, 22, 25]. A previous study from our group has shown that C/EBPα acts as a tumor suppressor gene by upregulating miR-223 in normal granulopoiesis [26]. In the present study, we show that ABR induces the transcription factor C/EBPα and thereby increases the expression of *G-CSF-R*, *M-CSF-R* and miR-223 (Figure 4A–4F) suggesting that ABR expression could have a significant role in myeloid differentiation and in leukemia through induction of the *CEBPA* gene and its target genes.

**Figure 8:**
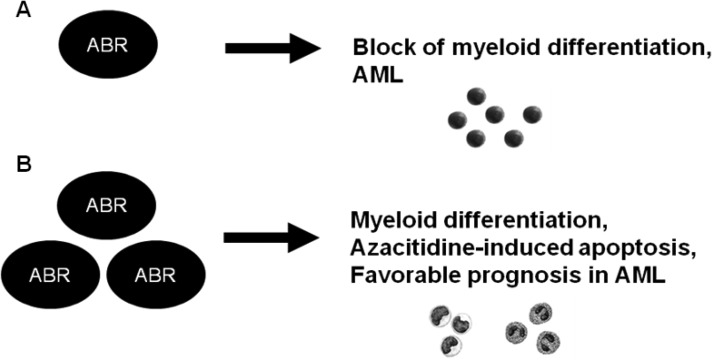
Schematic representation of a model for the role of ABR in normal myelopoiesis and in AML A knockdown of ABR expression resulted in block of myeloid differentiation. In accordance to this, ABR expression is reduced in AML (**A**). Conversely, ABR overexpression enhanced myeloid differentiation and azacitidine-induced apoptosis. A high *ABR* expression correlated with better outcome in AML (**B**).

Taken together, we discovered ABR as a new critical player in myeloid differentiation. We show that myeloid differentiation inducers lead to increased expression levels of the ABR and CEBPα targets M-CSF-R and G-CSF-R in human and mouse myelopoiesis. A reduction of ABR expression leads to decreased CEBPα levels and a minimized myeloid differentiation (Figure 8A). In AML, we observed a decreased ABR expression. Conversely, high ABR expression enhanced myeloid differentiation; correlated with a favorable prognosis in AML and increased azacitidine-induced apoptosis (Figure 8B). Further, a response to azacitidine treatment correlated with a high ABR expression. Our data indicate the functional role of ABR as a new player in myelopoiesis and AML. Because ABR has been shown to control innate immune response and ABR deletion has been found in several human cancers, targeted treatment that increase endogenous levels of ABR might provide novel therapeutic strategies to enhance the treatment response for patients not only restricted to AML, as the immune system is able to recognize and attack cancer cells in general.

## MATERIALS AND METHODS

### Human cell samples from AML patients and healthy donors

AML patient samples belonging to the cohort that received non-myeloablative hematopoietic stem cell (NMA-HSC) transplantation [27] and bone marrow samples from patients without any hematopoietic disease were obtained from University Hospital of Leipzig. Samples from azacitidine-treated AML patients were obtained from University Hospital of Halle. Azacitidine was administered as previously described [28]. The study protocols used for AML patient sample collection were approved by the ethics committees of the participating centres. All patients provided written informed consent in accordance with the Declaration of Helsinki. All samples were analysed by cytogenetic and molecular genetic analysis.

### Animals

C57Bl/6 mice were bred at the Animal Facility at the University of Leipzig, housed, treated and handled in accordance with the guidelines of the University Leipzig Animal Care Committee and the Regional Board of Animal Care for Leipzig.

### Cell culture and flow cytometry analysis

U937 cells were maintained in RPMI 1640 medium supplemented with 10% fetal bovine serum (FBS), 1% penicillin-streptomycin. For myeloid differentiation of U937 cells, 1x10^6^ cells were induced once with 10nM PMA (phorbol 12-myristate 13-acetate). For differentiation of ABR siRNA transfected U937 cells, 1nM PMA was used. Mouse bone marrow cells were obtained from tibiae and femora from C57Bl/6 wild-type mice and were seeded at 1x10^6^/mL in Dulbecco’s modified Eagle’s medium (DMEM) complemented with 10% FBS, 1% penicillin-streptomycin and dexamethasone (10^–8^ M). Differentiation of granulocytic and monocytic lineages were induced with G-CSF (1 ng/ml) or M-CSF (20 ng/ml), respectively. Treatment of the cells was done daily during the experimental process. Cell differentiation was assessed by flow cytometry analysis using PE-conjugated mouse anti-human CD11b antibody (BD Biosciences), FITC-conjugated rat anti-mouse F4/80 and FITC-conjugated rat anti-mouse Gr-1 (eBioscience). Apoptosis was measured with an Annexin V Apoptosis Detection Kit I (BD Bioscience) according to the manufacturer’s instructions. For Rac inhibition, NSC23766 (Selleckbiochem) was added daily to the cell culture medium.

### mRNA and miRNA detection by quantitative real-time PCR

Total RNA was isolated from cells using TRIzol reagent (Invitrogen). RNA (100 ng) was used to synthesize cDNA by reverse transcription. Equal amounts of cDNA were taken for a subsequent quantitative real-time PCR (qPCR) using the SYBR Green PCR kit (Applied Biosystems) as previously described [29]. The relative quantity of mRNA was determined by the comparative threshold cycle method using glyceraldehyde-3-phosphate dehydrogenase (GAPDH) or 18S ribosomal RNA expression for normalization. All reactions were performed in triplicates. Primer sequences are provided in Supplementary Table 1. MiRNA quantification was performed as previously described [29] by using RNU6B expression for normalization. Corresponding reverse transcription (RT) and qPCR primers for RNU6B and miR-223 were obtained from Applied Biosystems. All reactions were performed in triplicates.

### Immunoblot analyses

Immunoblot analyses were performed as previously described [29]. For ABR protein detection, a mouse monoclonal antibody anti-ABR (Abcam), and for C/EBPα protein detection, a rabbit monoclonal antibody anti-C/EBPα (Abcam) was used. Polyclonal rabbit anti-GAPDH (sc-25778; Santa Cruz Biotechnology) and b-tubulin (sc-9104) antibodies were used for normalization. The immunoreactivity was determined using an enhanced chemiluminescence method (Amersham Biosciences) according to the manufacturer’s instructions. The band intensities were quantified using ImageJ software (National Institute of Health, Bethesda, MD).

### Transfections

Transfection of pEYFP-ABR, pcDNA3.1-E2F1 and the according control vectors as well as ABR siRNA, CEBPα siRNA or control siRNA in U937 cells was performed via electroporation with a specific Nucleofactor Kit according to the manufacturer’s instructions (Lonza). 2 µg of DNA plasmid or 800 nM siRNA were used for each transfection. The pEYFP-ABR construct, as well as the empty vector pEYFP, were kindly provided by Professor Dr. N. Heisterkamp [23]. The siRNA for ABR and C/EBPα were purchased from Invitrogen. The siRNA sequences are provided in Supplementary Table 2.

### Lentiviral transduction

Lentiviral vector for EYFP-Abr in pCCL-cppt178-MNDU3 was a gift from Professor Dr. N. Heisterkamp (Addgene plasmid # 38155) [23]. 293TN cells were co-transfected using polyethylenimine (PEI) with either control vector or EYFP-ABR in pCCL-cppt178-MNDU3.Packaging plasmids psPAX2 (Addgene plasmid # 12260) and pMD2.G (Addgene plasmid # 12259) were gifts from Didier Trono. Virus-containing supernatants were collected at 24 and 48 hours after transfection and stored at -80°C. Lentiviral transduction of U937 cells was performed in 24-well culture dishes for 2 consecutive days.

### Statistical analysis

We used *Student t test* to determine the statistical significance of experimental results. A *P* value of .05 or less was considered significant. The results were represented as the mean ± standard deviation (SD) from at least three independent experiments. The AML patients that received NMA-HSC transplantation were dichotomized into *ABR* high and *ABR* low expressers using a median cut. For time-to-event analyses, we calculated survival estimates using the Kaplan–Meier method, and compared groups by the log-rank test [30]. Azacitidine-treated AML patients were divided into responders and non-responders, according to blast clearance (≤ 5%) in the bone marrow on day 15 after start of the first azacitidine treatment cycle [28]. Kruskal test was used for statistical evaluation of *ABR* expression.

## SUPPLEMENTARY MATERIALS TABLES




