# Supplementary material for: ABR, a novel inducer of transcription factor C/EBPα, contributes to myeloid differentiation and is a favorable prognostic factor in acute myeloid leukemia
Source: Oncotarget. 2017 Oct 26;8(61):103626–39. doi: 10.18632/oncotarget.22093 (PMC5732755; doi:10.18632/oncotarget.22093)
Supplement: Supplementary file 1 [file oncotarget-08-103626-s001.pdf]

ABR, a novel inducer of transcription factor C/EBPα, contributes to myeloid differentiation and is a favorable prognostic factor in acute myeloid leukemia

SUPPLEMENTARY MATERIALS

Supplementary Table 1: Primer sequences

	Primer sequence
h ABR forward	5′-agc cga gat atg agc ctg aa-3′
h ABR reverse	5′-cct cga tac ccc tct tct cc-3′
m ABR forward	5′-tga agg acg gct tcc tgg tgg a-3′
m ABR reverse	5′-tcg gct ctg tcc ttt gtt ggc t-3′
h CEBPA forward	5′-tgg aca aga aca gca acg ag-3′
h CEBPA reverse	5′-ttg tca ctg gtc agc tcc ag-3′
h GAPDH forward	5′-acc aca gtc cat gcc atc ac-3′
h GAPDH reverse	5′-tcc acc acc ctg ttg ctg ta-3′
h G-CSF-R forward	5′-acc tgg gca cag ctg gag tgg-3′
h G-CSF-R reverse	5′-cag gtc gct gtg agc tgg gtc tgg-3′
h M-CSF-R forward	5′-gtg gct gtg aag atg ctg aa-3′
h M-CSF-R reverse	5′-cct tcc ttc gca gaa agt tg-3′
m M-CSF-R forward	5′-gct cgg cca cta acg ccg aa-3′
m M-CSF-R reverse	5′-ttc atg gtg gcc gtg cgt gt-3′

Supplementary Table 2: siRNA duplex sequences

	siRNA sequence
h ABR siRNA	uac aaa gcg uuu guc gau aac uau a<br>uau agu uau cga caa acg cuu ugu a
h CEBPA siRNA	agc gca aca aca ucg cgg ugc gca a<br>uug cgc acc gcg aug uug uug cgc u

Supplementary Table 3: Comparison of clinical and molecular characteristics of patients with acute myeloid leukemia that received NMA-HSC transplantation with high versus low ABR expression

Characteristic	Low ABR (*n* = 31)	High ABR (*n* = 32)	*P* value
Median age, y (range)	61 (21–74)	61 (38–73)	.29
Gender (male/female), *n*	15/16	16/16	1
Median Hb, g/dL (range)	9.2 (5.8–14.9)	9.0 (4.3–15.7)	.79
Median PLT, 109 /L (range)	56 (2–179)	48 (1–192)	.48
Normal karyotype, *n*	13	13	1
CEBPA mut	3 (10%)	3 (10%)	1
FLT3-ITD	8 (26%)	5 (16%)	.53
high MN1	16 (48%)	15 (53%)	.80
de novo AML, *n*	22	20	.60

Abbreviations: Hb, hemoglobin; PLT, platelets; FLT3-ITD, internal tandem duplicaton of the FLT3 gene; MN, meningioma. The median expression value was used as a cut point. It was calculated based on the expression levels assessed by qPCR. *P*-values compare patients who have low ABR expression vetsus high ABR expression by log rank test.

Supplementary Table 4: Characteristics of healthy donors and AML patients who received NMA-HSC transplantation used to analyse ABR expression. See Supplementary_Table_4

Supplementary Table 5: Genetic and morphological characteristics of AML patients and healthy donors used for ABR expression according to treatment response to azacitidine

Patient	Patient type	FAB	Gender	Age (years)	Blast count (t0), %	Blast count (t15), %
	no AML	–	m	53	–	–
	no AML	–	–	–	–	–
	no AML	–	–	–	–	–
1	non-responder	M2	m	79	34	10
2	non-responder	unknown	m	62	75	75
3	non-responder	M2	m	63	24	90
4	non-responder	M4	f	68	69	21
5	non-responder	M7	f	70	70	67
6	non-responder	M1	f	70	70	80
7	non-responder	M2	m	66	80	60
8	non-responder	M1	m	73	75	90
9	non-responder	M4	f	62	90	35
10	non-responder	M4	m	68	60	70
11	non-responder	M5	f	66	83	unknown
12	non-responder	M4	m	72	57	70
13	non-responder	M1	f	72	80	50
14	non-responder	M4	m	73	81	12
15	non-responder	M2	m	65	64	11
16	responder	M2	m	77	20	≤ 5
17	responder	M1	m	78	25	≤ 5
18	responder	M4	m	70	80	≤ 5
19	responder	M5	f	75	70	≤ 5
20	responder	M0+M2	m	68	52	≤ 5
21	responder	M1	f	64	95	≤ 5

FAB, French-American-British; f, female; m, male; t, time in days.
