# Supplementary material for: ABR, a novel inducer of transcription factor C/EBPα, contributes to myeloid differentiation and is a favorable prognostic factor in acute myeloid leukemia
Source: Oncotarget. 2017 Oct 26;8(61):103626–39. doi: 10.18632/oncotarget.22093 (PMC5732755; doi:10.18632/oncotarget.22093)
Supplement: Supplementary file 2 [file oncotarget-08-103626-s002.docx]

**Supplementary Table 4: Characteristics of healthy donors and AML patients who received NMA-HSC transplantation used to analyse ABR expression**

| Patient | Karyotype | Age | Gender | %PB blasts | %BM blasts | FAB subtype | FLT3-ITD |
| --- | --- | --- | --- | --- | --- | --- | --- |
| 1 | CK | 51 | f | 63 | 80 | M1 | negative |
| 2 | NK | 52 | f | 68 | 95 | M1 | negative |
| 3 | del(5) | 62 | f | unknown | unknown | M2 | negative |
| 4 | NK | 66 | m | unknown | 31 | M5 | positive |
| 5 | CK | 27 | m | 60 | 64 | M4 | positive |
| 6 | CK | 61 | f | 8 | 75 | M4 | negative |
| 7 | CK | 73 | f | 82 | 86 | M2 | negative |
| 8 | NK | 52 | m | 32 | 61 | M2 | negative |
| 9 | NK | 61 | f | 15 | 61 | M4 | negative |
| 10 | 5q- | 48 | f | 48 | 94 | M1 | negative |
| 11 | NK | 58 | f | 33 | unknown | not classified | negative |
| 12 | NK | 63 | f | 92 | 85 | M2 | positive |
| 13 | NK | 70 | m | 50 | 84 | M2 | positive |
| 14 | NK | 67 | m | 2 | 27 | M2 | negative |
| 15 | CK | 72 | m | 86 | 75 | M2 | negative |
| 16 | CK | 60 | m | 18 | 40 | M4 | unknown |
| 17 | NK | 66 | m | 66 | 66 | M2 | negative |
| 18 | CK | 19 | m | 61 | 84 | M4 | positive |
| 19 | CK | 30 | f | 85 | 95 | M5 | positive |
| 20 | CK | 61 | f | 93 | 80 | M2 | positive |
| 21 | NK | 57 | f | 28 | 42 | M2 | negative |
| 22 | NK | 59 | m | 12 | 87 | M5 | negative |
| 23 | NK | 71 | m | 78 | 88 | M2 | negative |
| 24 | CK | 67 | m | 31 | unknown | not classified | negative |
| 25 | inv(16) | 46 | f | 46 | 40 | M4 | negative |
| 26 | CK | 67 | f | 92 | 95 | M2 | negative |
| 27 | NK | 62 | m | 77 | 80 | M1 | negative |
| 28 | CK | 20 | f | 33 | 45 | M4 | negative |
| 29 | inv(2) | 63 | m | 97 | 90 | M2 | negative |
| 30 | CK | 63 | m | 36 | 90 | M2 | negative |
| 31 | CK | 53 | f | 6 | 50 | M5 | positive |
| 32 | t(11;20) | 50 | f | 2 | 63 | M2 | negative |
| 33 | CK | 62 | f | 26 | 61 | M2 | unknown |
| 34 | NK | 65 | f | 44 | 60 | M2 | negative |
| 35 | NK | 58 | f | 41 | 85 | M4 | negative |
| 36 | CK | 64 | f | 11 | 45 | M6 | negative |
| 37 | t(X;19) | 66 | f | 8 | 25 | M7 | negative |
| 38 | NK | 68 | m | unknown | unknown | M2 | negative |
| 39 | NK | 72 | m | 0 | 51 | M2 | negative |
| 40 | CK | 66 | m | 62 | 87 | M1 | negative |
| 41 | unknown | 60 | f | 6 | 57 | M1 | positive |
| 42 | t(6;9) | 59 | f | 79 | 82 | M2 | positive |
| 43 | CK | 51 | f | 12 | 83 | M1 | negative |
| 44 | t(1;2) | 60 | m | 38 | 75 | not classified | negative |
| 45 | NK | 63 | m | 0 | 50 | M1 | unknown |
| 46 | CK | 60 | f | 2 | 60 | M2 | negative |
| 47 | NK | 66 | f | 7 | 34 | M4 | positive |
| 48 | CK | 67 | m | 39 | 92 | not classified | negative |
| 49 | CK | 52 | m | 37 | 10 | M1 | negative |
| 50 | der(1;7) | 59 | m | 20 | unknown | not classified | negative |
| 51 | NK | 59 | f | 3 | 26 | M5 | positive |
| 52 | NK | 63 | m | unknown | unknown | not classified | negative |
| 53 | CK | 48 | f | 21 | 50 | M7 | negative |
| 54 | CK | 62 | m | 12 | 30 | M2 | positive |
| 55 | t(11;19) | 64 | m | 4 | 44 | M4 | positive |
| 56 | NK | 50 | m | 86 | 85 | M0 | unknown |
| 57 | NK | 58 | m | 97 | 94 | M4 | negative |
| 58 | unknown | 37 | f | 90 | 90 | M1 | negative |
| 59 | NK | 61 | f | 34 | 30 | M1 | negative |
| 60 | unknown | 59 | m | unknown | 90 | M2 | positive |
| 61 | NK | 63 | m | 38 | 50 | M2 | negative |
| 62 | NK | 62 | m | 20 | 75 | M0 | negative |
| 63 | t(8;21) | 53 | f | 22 | 40 | M2 | negative |

Abbreviations: CK, complex karyotype; NK, normal karyotype; f, female; m, male; PB, peripheral blood; BM, bone marrow; FAB, French-American-British; FLT3-ITD, internal tandem duplication of FLT3 gene.
